# ATG-18 and EPG-6 are Both Required for Autophagy but Differentially Contribute to Lifespan Control in *Caenorhabditis elegans*

**DOI:** 10.3390/cells8030236

**Published:** 2019-03-12

**Authors:** Zsuzsanna Takacs, Katharina Sporbeck, Jennifer Stoeckle, Maria Jhaneth Prado Carvajal, Mona Grimmel, Tassula Proikas-Cezanne

**Affiliations:** 1 Department of Molecular Biology, Interfaculty Institute of Cell Biology, Eberhard Karls University, 72076 Tuebingen, Germany; zsuzsanna.takacs@imba.oeaw.ac.at (Z.T.); katharina.sporbeck@student.uni-tuebingen.de (K.S.); jennifer.stoeckle@uni-hohenheim.de (J.S.); maria-jhaneth.prado-carvajal@student.uni-tuebingen.de (M.J.P.C.); mona.grimmel@med.uni-tuebingen.de (M.G.); 2 International Max Planck Research School ‘From Molecules to Organisms’, Max Planck Institute for Developmental Biology and Eberhard Karls University, 72076 Tuebingen, Germany

**Keywords:** ATG-18, autophagy, EPG-6, GFP::LGG-1, lifespan, WIPI3, WIPI4

## Abstract

During macroautophagy, the human WIPI (WD-repeat protein interacting with phosphoinositides) proteins (WIPI1–4) function as phosphatidylinositol 3-phosphate effectors at the nascent autophagosome. Likewise, the two WIPI homologues in *Caenorhabditis elegans*, ATG-18 and EPG-6, play important roles in autophagy, whereby ATG-18 is considered to act upstream of EPG-6 at the onset of autophagy. Due to its essential role in autophagy, ATG-18 was found to be also essential for lifespan extension in *Caenorhabditis elegans*; however, this has not yet been addressed with regard to EPG-6. Here, we wished to address this point and generated mutant strains that expressed the autophagy marker GFP::LGG-1 (GFP-LC3 in mammals) and harbored functional deletions of either *atg-18* (*atg18(gk378)*), *epg-6* (*epg-6(bp242)*) or both (*atg-18(gk378);epg-6(bp242)*). Using quantitative fluorescence microscopy, Western blotting, and lifespan assessments, we provide evidence that in the absence of either ATG-18 or EPG-6 autophagy was impaired, and while *atg-18* mutant animals showed a short-lived phenotype, lifespan was significantly increased in *epg-6* mutant animals. We speculate that the long-lived phenotype of *epg-6* mutant animals points towards an autophagy-independent function of EPG-6 in lifespan control that warrants further mechanistic investigations in future studies.

## 1. Introduction

Macroautophagy (hereafter referred to as autophagy) is an evolutionarily conserved bulk degradation process across eukaryotes. It describes a cytoprotective mechanism during which cytoplasmic materials such as long-lived proteins, lipids, and damaged cell organelles are sequestered in autophagosomes. The engulfed material is subsequently delivered to the lysosome and degraded by lysosomal hydrolases [1]. Autophagy is regulated by the concerted action of autophagy related (ATG) proteins [2]. Initiation of autophagy under energy or nutrient deprivation conditions leads to the activation of ULK1 (UNC51-like kinase 1) in mammals, and its homologue UNC-51 (uncoordinated 51) in *Caenorhabditis elegans* [3]. Subsequently, ULK1 has been shown to phosphorylate members of the phosphatidylinositol 3-kinase class III (PI3KC3) complex that produces phosphatidylinositol 3-phosphate (PI3P) at initiation sites for autophagosome formation [2].

Newly produced PI3P is recognized by members of the conserved PROPPIN (β-propellers that bind phosphoinositides) protein family that include the four WIPI (WD repeat proteins interacting with phosphoinositides) proteins (WIPI1 through WIPI4) in mammals and the two members ATG-18 and EPG-6 in *C. elegans*, all of which are considered to function as PI3P effectors at the nascent autophagosome [4]. In mammals, PI3P-bound WIPI2B and WIPI2D have been found to specifically associate with the ATG16L complex, which in turn promotes the conjugation of LC3 (microtubule-associated protein 1A/1B-light chain 3; LGG-1 (LC3, GABARAP and GATE-16 family 1) in *C. elegans*) to phosphatidylethanolamine at the nascent autophagosome [5,6,7]. In *C. elegans*, this function is considered to be carried out by ATG-18 [3], which together with WIPI1 and WIPI2 belongs to one of the two paralogous groups of the PROPPIN family [5,8,9]. Together with WIPI3 and WIPI4, EPG-6 belongs to the other paralogous PROPPIN group, and in fact, overlapping functions of EPG-6 and WIPI4, both of which specifically associate with ATG2 (ATG-2 in *C. elegans*), control the size of forming autophagosomes [7,10].

In *C. elegans*, ATG-18 is required for lifespan control, as mutant animals deficient for wild-type ATG-18 function display features of accelerated ageing and exhibit a short-lived phenotype [11,12,13,14]. Likewise, ATG-18 was found to be required for longevity [3] promoted by dietary restriction, inhibition of insulin-mediated TOR (target of rapamycin) signaling or germline ablation [15,16,17,18,19]. However, the role of ATG-18 upon starvation has not yet been investigated. The role of EPG-6 function in starvation has been assessed, and it was shown that in the absence of food, *C. elegans* L1 larvae deficient for EPG-6 function show a reduced capacity to survive starvation [10]. However, the role of EPG-6 in an adult lifespan control is unknown.

In this study, we aimed to contribute to the further characterization of the role of ATG-18 (autophagy related 18) and EPG-6 (ectopic P granules 6) in autophagy in *C. elegans*, from embryo to the larval stages L1 through L4, to further investigate the capacity of *atg-18* and *epg-6* mutant animals to recover and survive L1 starvation periods, and finally, to assess their adult lifespan. To assess autophagy, we quantified the number of autophagic structures detected as fluorescent GFP::LGG-1 puncta, the GFP-tagged autophagy marker LGG-1 (LC3 in mammals), throughout development of *C. elegans*, and in addition, we assessed the protein levels of GFP::LGG-1 by Western blotting. We show that GFP::LGG-1 puncta aberrantly accumulated in all larval stages (L1–L4), and that GFP-cleavage from GFP::LGG-1, indicative for the autophagic flux in the presence of lysosomal inhibitors, was reduced. These results underline the requirement of both ATG-18 and EPG-6 in autophagosome formation and autophagic flux in *C. elegans*. In contrast, when we assessed the capacity to recover from L1 starvation periods, we found that ATG-18 deficient strains were unable to survive starvation, whereas the EPG-6 deficient strain showed a less prominent decrease in the survival rate when compared to wild-type *C. elegans* (N2). Moreover, whereas adult *C. elegans*’ lifespan significantly decreased in the ATG-18 deficient strain, lifespan significantly increased in the absence of functional EPG-6. Hence, *atg-18* mutant animals exhibit short-lived and *epg-6* mutant animals a long-lived phenotype. Based on our combined results, we suggest that the long-lived phenotype in the absence of EPG-6 should be due to an autophagy-independent function of EPG-6. 

## 2. Materials and Methods

### 2.1. Strain Maintenance

*Caenorhabditis elegans* strains were grown on standard nematode growth medium (NGM) (3 g/L NaCl, 17 g/L agar, 2.5 g/L peptone, 5 mg/L cholesterol, 1 mM MgSO_4_, 1 mM CaCl_2_, 2.5% *v*/*v* KPO_4_-buffer pH 6) (KPO_4_ buffer: 0.87 M KH_2_PO_4_, 0.13 M K_2_HPO_4_) plates seeded with *E. coli* OP50 at 15 °C using standard techniques [20]. All experiments were performed at 20 °C. The following strains were provided by the CGC (Caenorhabditis Genetics Center, University of Minnesota, Minnesota, MN, USA) (Table S1): N2 Bristol wild isolate was used as wild-type (WT); the VC893 strain (*atg-18(gk378) V*) was used to assess animals harboring a functional deletion of 666 bp in *atg-18* where exon 1 to 3 are missing [12]; the HZ1690 strain (*epg-6(bp242) III; him-5(e1490) V*) harbors a functional deletion of *epg-6* due to a C/T substitution in exon 3 resulting in a premature stop [10], the DA2123 strain (*adIs2122* [*lgg-1p::GFP::lgg-1 + rol-6*(*su1006*)]) constitutively expressed the autophagy marker GFP::LGG-1 [21,22]. The following strains were generated for this study: a double-mutant strain deficient for both ATG-18 and EPG-6 function (*atg-18(gk378) V*; *epg-6(bp242) III*; *him-5(e1490) V*); a reporter strain deficient for ATG-18 function and expressing GFP::LGG-1 (*atg-18(gk378) V*; *lgg-1::GFP+rol-6(su1006)*); a reporter strain deficient for EPG-6 function and expressing GFP::LGG-1 (*epg-6(bp242) III*; *him-5(e1490) V*; *lgg-1::GFP+rol-6(su1006)*). Each strain was backcrossed at least three times (Table S1). 

### 2.2. Bacteria Culture as Food Source

Bacterial cultures using *E. coli* OP50 (from CGC) were prepared overnight at 37 °C, 180 rpm in liquid LB medium (10 g/L tryptone, 5 g/L yeast extract, 5 g/L NaCl). Bacterial cultures were always prepared freshly from glycerol stocks and then seeded onto NGM plates.

### 2.3. Generation of Male Stocks by Heat Shock

Wild-type *C. elegans* male stocks were purchased from CGC (Caenorhabditis Genetics Center, University of Minnesota, Minnesota, MN, USA). For all other strains, males were generated by heat shock by incubating 20–30 hermaphrodites per 6-cm plate at 30 °C for 6 h, which were then transferred back to 20 °C, and incubated for 3 days. After 3 days, males were used for crossing with L4 hermaphrodites of the same strain.

### 2.4. Crossing Strains

To generate GFP::LGG-1 expressing strains carrying the desired mutations in *atg-18* and/or *epg-6*, an *adIs2122* extrachromosomal transgene expressing DA2123 strain was used for crossing (Table S1). The adIs2122 transgene encoded an N-terminal GFP::LGG-1 fusion protein. A 1.7 kb fragment of the *lgg-1* promoter was cloned upstream of the GFP coding sequence, and a 2.2 kb fragment of the lgg-1 coding sequence was cloned downstream of the GFP sequence [11]. To remove background mutations, each strain was backcrossed with wild-type worms at least 3 times. For backcrossing, hermaphrodites carrying the allele of interest were crossed with wild-type (N2) males. Eight L4 hermaphrodites and at least 15–20 males were transferred to a small agar piece (about 1/3 of a 6-cm NGM agar plate) covered with *E. coli* OP50. The rest of the agar was removed using a sterile spatula before transferring the worms. The plate was incubated at 20 °C overnight, and the next morning, the hermaphrodites were transferred singly onto NGM plates covered with *E. coli* OP50. The plates were incubated at 20 °C, and after 3 days the progeny was inspected for males. As the presence of males represents successful mating, plates with the highest ratio of males were chosen for further analysis. From the F1 progeny, heterozygous L4 hermaphrodites were singled again, and incubated at 20 °C. After 2 days, the F1 hermaphrodites were lysed singly, and genotyped by single worm lysis (SWL)-PCR. From a plate, where the F1 hermaphrodite was heterozygous, F2 L4 hermaphrodites—from which 25% should be homozygous mutant—were transferred singly to fresh plates and incubated at 20 °C. After 2 days, the F2 hermaphrodites were genotyped by single worm PCR. The plates where the F2 hermaphrodite was homozygous mutant was kept, and F3 worms were genotyped with single-worm PCR again. When all F3 worms were homozygous mutant, the population was considered as backcrossed once. For the generation of double-mutant strains, crossing was done as described above, except that the hermaphrodites were not crossed with wild-type males, but with males carrying the allele of interest. 

### 2.5. Genotyping by Single-Worm Lysis PCR

For single worm lysis (SWL), PCR worms were lysed singly in 10 μL single-worm lysis-buffer (SWL-buffer) (0.05 M KCl, 2.5 mM MgCl_2_·6H_2_O, 0.01 MTris, 0.45% Tween-20, 0.45% Triton-X) supplemented with 1 μL proteinase K (NEB, P8102S), and incubated at 65 °C for 1 h, then at 95 °C for 20 min. The DNA of single worms was used for PCR. For one PCR reaction, 25 units of Taq DNA polymerase (NEB, M0273) with standard Taq buffer (NEB, M0273L), 200 μM dNTP (NEB, N0446S), 0.5 μM of both primers (Sigma–Aldrich, Munich, Germany), and 5 μL DNA was used. For genotyping of *epg-6(bp242)* tetra-primer amplification-refractory mutation system PCR (tetra-primer ARMS-PCR) was applied [23]. For ARMS-PCR, the inner primers were used at 1 μM, the outer primers at 0.1 μM final concentration. 

The following primers were used: 

*atg-18(gk378)* forward primer (5′-3′): TGCAATCTTCCAAACATACGA; 

*atg-18(gk378)* reverse primer (5′-3′): CCAAAAATGCCACCAAGCTA; 

*epg-6(bp242)* forward inner primer: GGACGTATCCAGATGATATTAAACAAAGCC; 

*epg-6(bp242)* reverse inner primer (T allele): GGATTTGAACGAATATCTTCCGAGCA; 

*epg-6(bp242)* forward outer primer (5′-3′): CGGACCTATTACAAACATCCACGTATCA; 

*epg-6(bp242)* reverse outer primer (5′-3′): GTGCATTTAGATGCATAATTTGAACGGA.

### 2.6. Brood-Size Assay

Stage L4 hermaphrodites were transferred singly to NGM plates seeded with *E. coli* OP50, and incubated at 20 °C. Hermaphrodites were transferred to a new plate once a day, until they laid eggs. Eggs were counted right after the hermaphrodite was transferred to a new plate, and L4/adult progeny was counted 2–3 days later. The brood size was determined as the number of progeny that reached L4/adult stage.

### 2.7. Egg Isolation Using Hypochlorite Treatment 

Gravid adult worms were grown in high density and used for isolation of eggs as follows. Animals were washed off NGM plates with H_2_O, centrifuged (2 min, 2000 rpm, RT), supplemented with bleach solution (hypochlorite treatment), and shook until the worms lysed. Subsequently, tubes with lysed worms were filled with M9 buffer (3 g/L KH_2_PO_4_, 6 g/L Na_2_HPO_4_, 5 g/L NaCl, 1mM MgSO_4_) and centrifuged (2000 rpm, 2 min). Finally, eggs were washed three times with M9 buffer. 

### 2.8. L1 Survival Assay

Embryos were prepared using hypochlorite treatment (2–4% NaOCl, 1.5 M NaOH) of gravid adults, and collected in M9 buffer (3 g/L KH_2_PO_4_, 6 g/L Na_2_HPO_4_, 5 g/L NaCl, 1 mM MgSO_4_) (1000 embryos/mL). The liquid culture was incubated without food at 20 °C, shaking (180 rpm). The first week every day, after that, 3 times a week 50 μL aliquots were placed on NGM plates seeded with *E. coli* OP50. The number of L1 larvae was counted right away. After 2–4 days at 20 °C, the numbers of larvae that could develop further and were able to pass the L1 larval stage were counted. The percentage of worms that survived starvation and could recover in the presence of food was calculated.

### 2.9. Lifespan Assay 

Lifespan assays were performed at 20 °C. Synchronous animal populations were generated by hypochlorite treatment (2–4% NaOCl, 1.5 M NaOH) of gravid adults. Worms were allowed to grow on normal NGM plates seeded with *E. coli* OP50 until the L4 larval stage. Larval stage L4 animals were transferred to NGM plates supplemented with 5 mg/L FUDR (5-Fluoro-2′-deoxyuridin, Sigma–Aldrich, F0503). Twenty animals were transferred to one plate, and 3–5 plates were counted for each strain in one set. Animals were counted every 2–3 days and scored as dead when they stopped responding to gentle prodding with a platinum wire. Dead animals were removed from the plates. Animals dried out on the walls were censored. When lifespan assays were conducted in the absence of FUDR, animals were counted and transferred to fresh NGM plates every day. Statistical analyses of the lifespan assays were done with the “Online Application for the Survival Analysis of Lifespan Assays Performed in Aging” software using basic survival analysis [24].

### 2.10. Lifespan Assay with RNAi Treatment

For RNAi treatments *E. coli* HT115 expressing an empty pL4440 plasmid or pL4440 containing the EPG-6 targeting RNAi construct were seeded on LB agar plates containing 12.5 µg/mL tetracycline (Sigma–Aldrich, 87128) and 50 µg/mL ampicillin (Applichem, Darmstadt, Germany; No. A0839) and incubated overnight at 37 °C. For liquid culture, the bacteria were grown in LB medium supplemented with 50 µg/mL ampicillin for 10–16 h at 250 rpm and 37 °C. For the lifespan assays, the liquid culture was seeded onto NGM plates supplemented with 1 mM IPTG (Isopropyl β-d-1-thiogalactopyranoside, Sigma–Aldrich, I6758), 25 µg/mL carbenicillin (Applichem, Darmstadt, Germany; No. A1491), and 5 mg/L FUDR. The plates were incubated at room temperature overnight to allow induction of the RNAi expression by IPTG. Animals were synchronized as described above (see Section 2.9) before transfer to the plates covered with bacteria expressing EPG-6 targeting or control RNAi constructs. The animals were incubated at 20 °C throughout the experiment and transferred to fresh RNAi plates every 3–5 days. Viability was scored every second to third day, as described for the standard lifespan assay (see Section 2.9). The EPG-6 targeting construct was purchased from Source Bioscience (clone ID: DFCI11006A10) as part of the RNAi library of Marc Vidal’s lab (Dana–Farber Cancer Institute, Boston, MA, USA) [25]. The empty pL4440 vector expressing the HT115 strain was a gift from Della David (German Center for Neurodegenerative Diseases, Tübingen, Germany).

### 2.11. Light Microscopy and Length Determination of C. elegans Strains

Animals were cultured on NGM plates supplemented with 5 mg/L FUDR (5-Fluoro-2′-deoxyuridin, Sigma–Aldrich, F0503) and imaged at 14 days of adulthood. Images were taken using a Nikon AZ100 microscope at 1.2× magnification. The length of the animals was determined by approximation of the nematode from the tip of the mouth to the end of the tail using the “polyline” function of NIS elements v4.

### 2.12. GFP::LGG-1 Puncta Assessments

Eggs were isolated from adult animals using hypochlorite treatment (2–4% NaOCl, 1.5 M NaOH) of gravid adults. For the microscopy of embryos, eggs were incubated in M9 buffer (3 g/L KH_2_PO_4_, 6 g/L Na_2_HPO_4_, 5 g/L NaCl, 1 mM MgSO_4_) at 20 °C, and prepared for microscopy right after egg isolation (0 h), or 4 h later (4 h). For the microscopy of L1, L2, L3, L4, and adult worms, eggs were seeded on standard NGM plates with *E. coli* OP50, and incubated for 6, 28, 52, and 76 h at 20 °C. For mounting on microscopy slides an agar pad was used, and worms were anaesthetized using 0.1 M NaN_3_. Fluorescent (GFP) and DIC images were taken using an Axiovert 200M (Carl Zeiss Microscopy GmbH, Munich, Germany) and an EC Plan-Neofluar 100×/1.30 Oil M27 objective (embryos) or a Plan-Apochromat 63×/1.40 Oil Ph 3 objective (larvae, adult). Images were quantified using the stress granule counter plugin of ImageJ. To quantify puncta formation in embryos, we divided the embryos into three groups based on their developmental stage: early stage embryos (until the beam stage), and late stage embryos (after 1.5-fold until 3-fold). In L1–L4 larvae and adults, puncta formation was measured in the head and in the seam cells. In the case of the seam cells inclusion, count refers to inclusion count/cell. In the case of the head, the whole head was analyzed until the posterior bulb of the pharynx.

### 2.13. Western Blotting

For Western blot analysis of *gfp::lgg-1 (adIS2122)* expressing starved L1, larvae eggs were isolated from gravid adults using hypochlorite treatment (2–4% NaOCl, 1.5 M NaOH), and 20,000 eggs were incubated in 10 mL M9 buffer (3 g/L KH_2_PO_4_, 6 g/L Na_2_HPO_4_, 5 g/L NaCl, 1 mM MgSO_4_) with and without 1 mM NH_4_Cl at 20 °C, 180 rpm overnight (16 h). After overnight incubation, the worms were centrifuged (2000 rpm, 3 min), transferred into Eppendorf tubes, centrifuged again (2000 rpm, 5 min), and the supernatant was removed. To extract the proteins 100 μL hot 2× Laemmli buffer (50 mM Tris pH 6,8; 1.25 mM EDTA, pH 8,0; 12.5% glycerol; 20 g/L SDS; 50 mM DTT; 2.5% β-Mercaptoethanol; 0.25 g/L Bromphenol blue) was added to the worms, and the DNA was sheared using a 23G needle. Samples were boiled for 10 min. Equal volumes (80 μL) of the extracts were loaded on SDS-PAGE gels, and after separation, proteins were blotted onto PVDF membranes (Millipore, Merck KGaA, Darmstadt, Germany, Cat. No. IPVH00010). The anti-GFP antibody (Roche, Merck KGaA, Darmstadt, Germany, Cat. No. 11814460001) was used to detect GFP-LGG-1. Anti-α-tubulin antibody (Sigma–Aldrich, Merck KGaA, Darmstadt, Germany, Cat. No. T5168) was used as loading control. Enhanced chemiluminescence (ECL) was performed using Immobilon Western Chemiluminescent HRP Substrate (Millipore, Merck KGaA, Darmstadt, Germany, Cat. No. WBKLS0100). Enhanced chemiluminescence (ECL) was detected by the Fusion SL4 device, and quantification was performed using the FUSION-CAPT Advance Software (version 16.09b, Vilber Lourmat, Eberhardzell, Germany). Background levels were removed by the rolling ball method. The results were further analyzed in Microsoft Excel, where the “Volume” values were used to compare protein levels. Total GFP::LGG-1 (GFP::LGG-1-I + GFP::LGG-1-II) was normalized over tubulin, and the ratio of cleaved GFP was calculated by dividing cleaved GFP levels by total GFP levels (GFP::LGG-1 + GFP::LGG-1-II + cleaved GFP). 

### 2.14. Statistical Analysis

Statistical analyses were carried out using SPSS Statistics Version 25.0 (IBM, Ehningen, Germany). The data distribution was tested using the Shapiro–Wilk test. If the data were normally distributed, group differences were analyzed using one-sided ANOVA and subsequent Tukey’s post-hoc test, as indicated in the figure legend. When data were not normally distributed, the groups were analyzed using a Kruskal–Wallis test with sequential Dunn–Bonferroni to test for significances between the strains. Statistical analysis for Western blot results when only two strains were compared was carried out using a two-tailed Student’s *t*-test. 

## 3. Results

**Figure 1:**
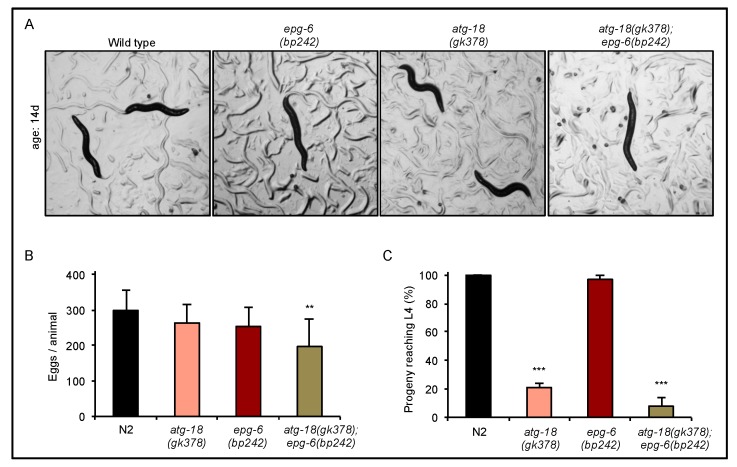
Viability and brood size decreased in the absence of functional autophagy related 18 (ATG-18). (**A**) Light microscopy images of *Caenorhabditis elegans* wild-type (N2), *atg-18(gk378)*, *epg-6(bp242)*, and *atg-18(gk378);epg-6(bp242)* strains were taken using a 1.2× objective and representative images are shown, *n* ≥ 9. (**B**) Single hermaphrodites were transferred to nematode growth medium (NGM) plates and the total number of eggs during the reproductive period per hermaphrodite was counted (*n* > 4). (**C**) The number of eggs hatching and reaching the larval stage 4 (L4)/adult stage was counted, and the percentages, based on the total egg number, was calculated. Mean values (+STDEV) of the number of eggs (**B**) and the brood size (**C**) are shown, and significant differences compared to wild-type indicated ** *p* < 0.01, *** *p* < 0.001, one-way ANOVA.

To investigate the role of ATG-18 and EPG-6 during autophagy, starvation, and lifespan in *C. elegans*, we characterized strains carrying loss-of-function mutations of *atg-18* (*atg-18(gk378)*) or *epg-6* (*epg-6(bp242)*), that were employed in previous studies [10,12], and using both of these strains, we generated a double mutant strain for *atg-18* and *epg-6* (*atg-18(gk378);epg-6(bp242)*) (Table S1). As already observed for *atg-18(gk378)* and *epg-6(bp242)* [10,12] mutant strains, also the *atg-18(gk378);epg-6(bp242)* strain superficially displayed a wild-type phenotype as assessed by light microscopy (Figure 1A) and length measurement of adult animals from the same age (data not shown). Next, we assessed the brood size of *atg-18(gk378)*, *epg-6(bp242)*, and *atg-18(gk378);epg-6(bp242)* strains in comparison to wildtype *C. elegans* (N2) (Figure 1B,C).

We observed that although the number of eggs laid by wild-type and mutant hermaphrodites differed significantly only in the *atg-18(gk378);epg-6(bp242)* double mutant (Figure 1B), and most of the *atg-18(gk378)*, *epg-6(bp242),* and *atg-18(gk378);epg-6(bp242)* eggs could hatch, the brood size (progeny that reached L4 larval or adult stage) of *atg-18(gk378)* and *atg-18(gk378);epg-6(bp242)* mutants significantly decreased (Figure 1C). In line with previous reports [10,11], our results show that loss of ATG-18 causes lethality at an early developmental stage, which is even higher in the absence of both ATG-18 and EPG-6, suggesting that ATG-18 and EPG-6 are essential survival factors under fed conditions. Of note, we did not observe a difference in brood size when comparing wild-type and *epg-6(bp242)* animals (Figure 1C).

**Figure 2:**
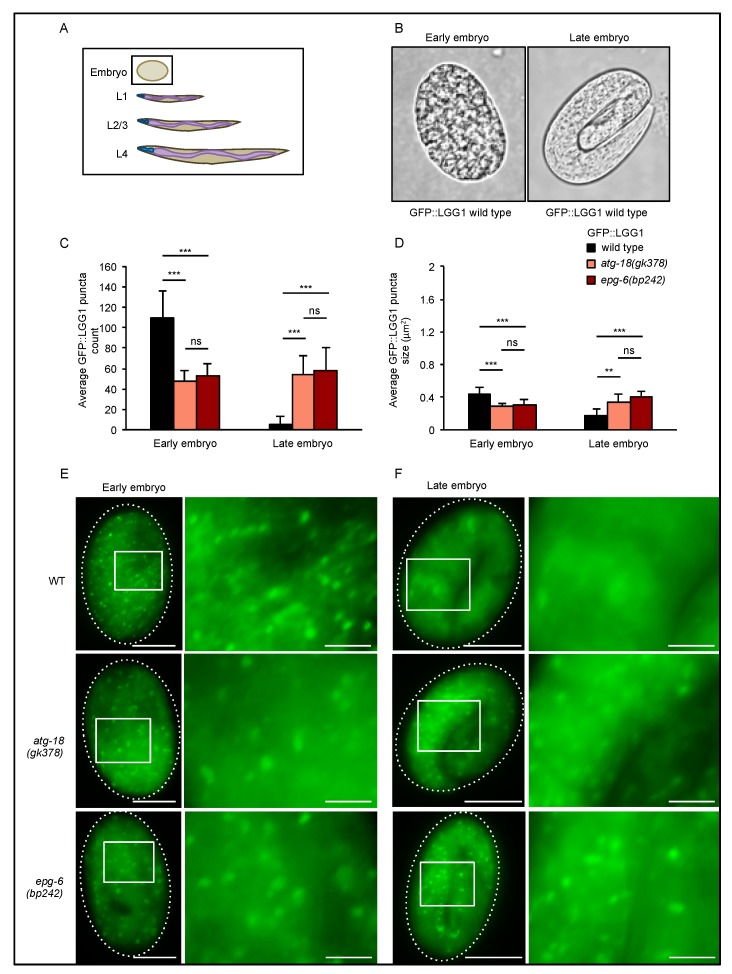
GFP::LGG-1 puncta formation decreased in early embryos, but enlarged puncta accumulate in late-stage embryos of *atg-18* and *epg-6* mutant animals. GFP::LGG-1 puncta formation was analyzed in wild-type, *atg-18(gk378),* and *epg-6(bp242)* embryos expressing the *adIS2122* transgene. The scheme in (**A**) indicates the developmental stage where GFP::LGG-1 puncta assessments were conducted, and the images in (**B**) show early- and late-stage embryos as examples. Fluorescence microscopy images were quantified using the stress granule counter plugin of ImageJ. The average number (**C**) and the average size (**D**) of GFP::LGG-1 puncta were calculated and representative images are shown (**E**,**F**). Scale bar: 20 μm. Significances were calculated using the Kruskal–Wallis test, ns: not significant, ** *p* < 0.01, *** *p* < 0.001. For more information, see Data S1.

**Figure 3:**
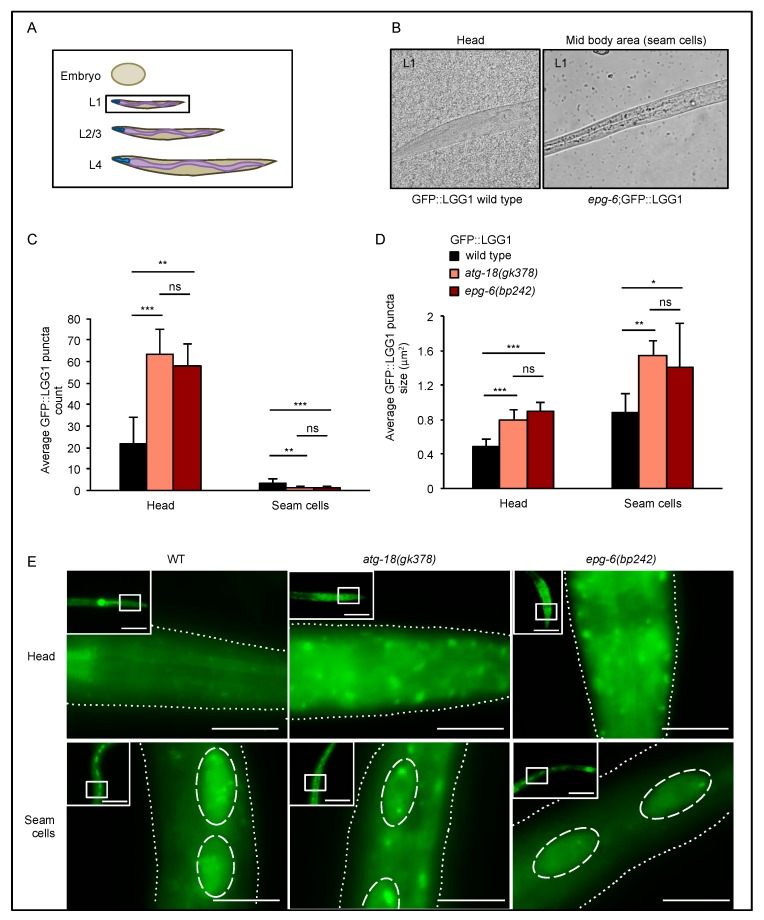
GFP::LGG-1 puncta accumulated in L1 larvae. GFP::LGG-1 puncta formation was analysed in the head and seam cells of wild-type, *atg-18(gk378),* and *epg-6(bp242)* L1 larvae expressing the *adIS2122* transgene. The scheme in (**A**) indicates the developmental larval stage (L1) where GFP::LGG-1 puncta assessments were conducted, and the images in (**B**) show head and mid-body regions, the latter with seam cells, as examples. Fluorescence microscopy images were quantified using the stress granule counter plugin of ImageJ. The average number (**C**) and size (**D**) of GFP::LGG-1 puncta were calculated. Representative images are shown (**E**). Scale bar: 20 μm. Significances calculated using the Kruskal–Wallis test, ns: not significant, * *p* < 0.05, ** *p* < 0.01, *** *p* < 0.001. For more information, see Data S1.

**Figure 4:**
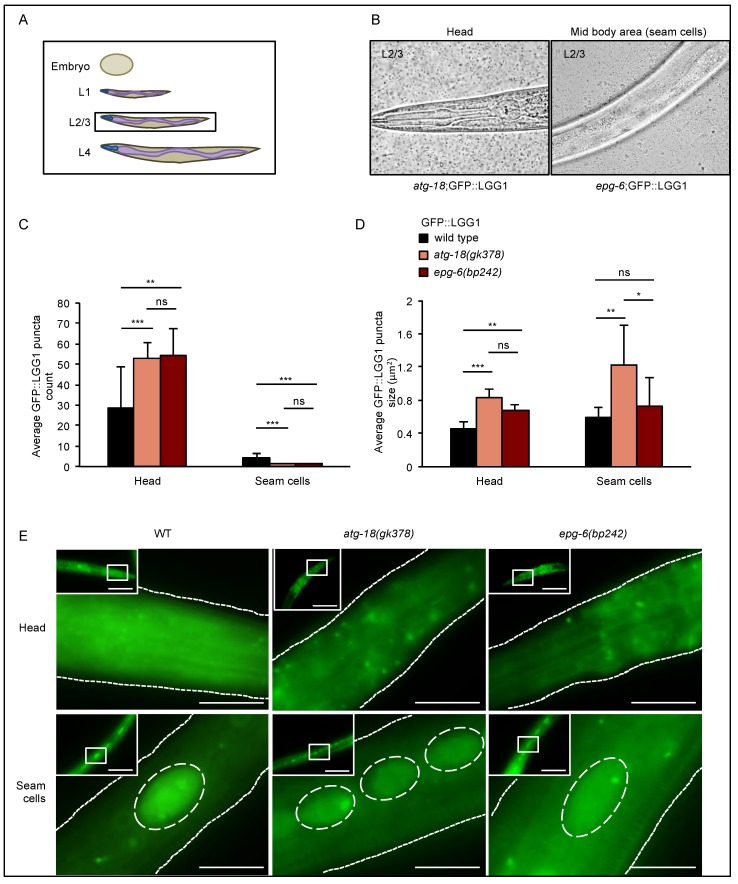
GFP::LGG-1 puncta accumulated in L2/3 larvae in the head but decreased in numbers in seam cells. GFP::LGG-1 puncta formation was analysed in the head and seam cells of wild-type, *atg-18(gk378),* and *epg-6(bp242)* L2/3 larvae expressing the *adIS2122* transgene. The scheme in (**A**) indicates the developmental larval stage (L2/3) where GFP::LGG-1 puncta assessments were conducted, and the images in (**B**) show head and mid-body regions, the latter with seam cells, as examples. Fluorescence microscopy images were quantified using the stress granule counter plugin of ImageJ. The average number (**C**) and size (**D**) of GFP::LGG-1 puncta were calculated. Representative images are shown (**E**). Scale bar: 20 μm. Significances calculated using the Kruskal–Wallis test, ns: not significant, * *p* < 0.05, ** *p* < 0.01, *** *p* < 0.001. For more information, see Data S1.

**Figure 5:**
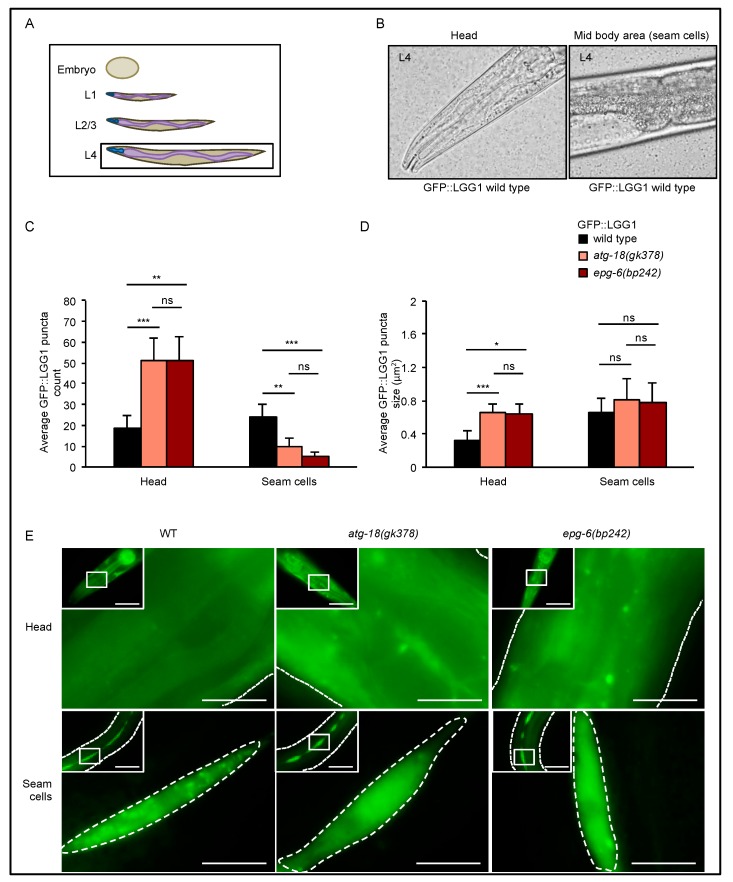
GFP::LGG-1 puncta accumulated in L4 larvae in the head but decreased in numbers in seam cells. GFP::LGG-1 puncta formation was analysed in the head and seam cells of wild-type, *atg-18(gk378),* and *epg-6(bp242)* L4 larvae expressing the *adIS2122* transgene. The scheme in (**A**) indicates the developmental larval stage (L4) where GFP::LGG-1 puncta assessments were conducted, and the images in (**B**) show head and mid-body regions, the latter with seam cells, as examples. Fluorescence microscopy images were quantified using the stress granule counter plugin of ImageJ. The average number (**C**) and size (**D**) of GFP::LGG-1 puncta were calculated. Representative images are shown (**E**). Scale bar: 20 μm. Significances calculated using the Kruskal–Wallis test, ns: not significant, * *p* < 0.05, ** *p* < 0.01, *** *p* < 0.001. For more information, see Data S1.

**Figure 6:**
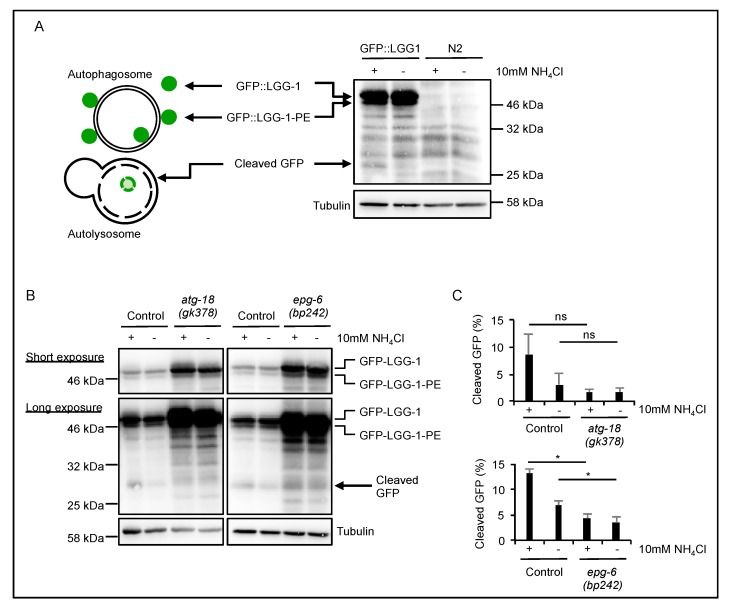
Western blot analysis of starved L1 animals revealed a block in autophagic flux upon loss of ATG-18 or EPG-6 function. The scheme indicates that GFP::LGG-1 localizes both to the outside and the inside of the autophagosome, hence become degraded after the fusion of the autophagosome with the lysosome (**A**, left). Cytosolic GFP-LGG-1 as well as membrane-bound GFP::LGG-1-PE and cleaved GFP is detected by Western blotting using GFP::LGG-1 expressing strains along with wild-type N2 control (**A**, right). Cleaved GFP, which can also be visualized by Western blotting, accumulates upon lysosomal inhibition due to a decelerated degradation using NH_4_Cl (**A**, right). L1 animals of wild-type (Control), *atg-18(gk378)*, and *epg-6(bp242)* expressing the *adIS2122* transgene were starved overnight in the presence (+) or absence (-) of NH_4_Cl, and proteins were extracted for anti-GFP Western blotting (**B**). A representative short exposure is shown and GFP::LGG-1 and GFP::LGG-1-PE migrations are indicated (**B**, upper panel). Likewise, a longer exposure is shown and the migration of cleaved GFP indicated (**B**, lower panel). Protein extracts from equal numbers of L1 larvae were used for anti-GFP western blotting and the appearance of cleaved GFP quantified, *n* = 3 (**C**). Significances calculated using two-tailed Student’s *t*-test, * *p* < 0.05.

Similar to assessing autophagy using LC3 in mammalian systems [26,27], the number of autophagic structures can be measured in *C. elegans* by visualizing the localization of membrane-bound, autophagosomal GFP-tagged LGG-1 (GFP::LGG-1) (GFP-LC3 homologue), which is detected as a punctate structure using fluorescent microscopy [21]. To generate GFP::LGG-1 expressing strains deficient for ATG-18 or EPG-6, we crossed the *adIS2122* strain (DA2123), which encodes an N-terminal GFP::LGG-1 fusion protein, with *atg-18(gk378)* or *epg-6(bp242)* mutant strains. Next, we quantified GFP::LGG-1 puncta formation (Data S1, Figure 2, Figure 3, Figure 4, Figure 5 and Figure 6) in early (up to comma stage) and late (after 1.5-fold until 3-fold) stage embryos (Figure 2), in the larval stages L1 (Figure 3), L2/L3 (Figure 4), and L4 (Figure 5) under nutrient-rich conditions, hence under basal autophagy conditions.

In early stage embryos (Figure 2A,B left panel), we found that both GFP::LGG-1 puncta number (Figure 2C, left panel) and size (Figure 2D, left panel) significantly decreased in the absence of ATG-18 and EPG-6 (representative images displayed in Figure 2E). As expected, in wild-type late-stage embryos, the number and size of GFP::LGG-1 puncta are significantly reduced when compared to early embryos of wild-type animals [28], but this was not observed in the absence of ATG-18 or EPG-6 (Figure 2C–F), suggesting that autophagic degradation is impaired and irregular autophagosomal structures accumulate.

During larval development, GFP::LGG-1 puncta formation was analyzed in the head and seam cells (Figure 3, Figure 4 and Figure 5). Enlarged GFP::LGG-1 puncta accumulated in the head of *atg-18(gk378);adIS2122* and *epg-6(bp242);adIS2122* L1 larvae (Figure 3C,D left panels, 3E upper panel). In seam cells, we also observed enlarged GFP::LGG-1 puncta but less in numbers (Figure 3C,D right panels, 3E lower panel). This result further suggests, that such enlarged GFP::LGG-1 puncta in both head and seam cells are due to impaired autophagic degradation and the accumulation of irregular autophagosomal structures in the absence of ATG-18 and EPG-6. 

In L2/L3 larvae, GFP::LGG-1 puncta formation was very similar to L1 larvae: enlarged autophagic structures accumulated in the head, whereas a reduced number of autophagic structures with aberrant size was observed in the seam cells of *atg-18(gk378);adIS2122* and *epg-6(bp242);adIS2122* L2/L3 larvae (Figure 4C–E). Accumulation of enlarged puncta in the head could also be observed in *atg-18(gk378);adIS2122* and *epg-6(bp242);adIS2122* L4 larvae (Figure 5C–E). Although in the seam cells of *atg-18(gk378);adIS2122* and *epg-6(bp242);adIS2122* L4 larvae, GFP::LGG-1 puncta were not enlarged any more, and puncta count was still significantly decreased (Figure 5C–E). Likewise, a significant reduction in the number of GFP::LGG-1 puncta was observed in adult seam cells of these mutant strains, and also an increase of enlarged GFP::LGG-1 puncta in the head of adult worms (Figure S1).

In summary, aberrantly enlarged GFP::LGG-1 decorated autophagic structures were observed during larval development (L1–4) in the head of *atg-18* and *epg-6* mutants (Figure 3, Figure 4 and Figure 5), suggesting a block in the autophagic flux in the absence of ATG-18 or EPG-6. Of note, in seam cells, GFP::LGG-1 puncta were almost non-visible in nutrient-rich conditions; however, they become visible, e.g., in ATG mutant strains due to a block in autophagy [21,28], or during challenging conditions, such as starvation, due to the induction of autophagy [21]. In our case, and according to previous findings [10,11], we suggest that the decrease in GFP::LGG-1 puncta numbers in seam cells (Figure 3, Figure 4 and Figure 5) likewise suggest that autophagy is impaired in the *atg-18* and *epg-6* mutant strains.

The autophagic flux can be assessed by measuring lipidation of GFP::LGG-1 by Western blotting [21,27] where non-lipidated, cytosolic GFP-LGG-1 is distinguished from GFP-LGG-1 conjugated to phosphatidylethanolamine (GFP-LGG-1–PE) at autophagosomal membranes based on different migration patterns (Figure 6A). Using GFP::LGG-1 also offers the possibility to compare the levels of cleaved GFP. GFP is cleaved from LGG-1 after the autophagosome fuses with the lysosome, and proteins enclosed in the autolysosome are degraded. In the presence of ammonium chloride (NH_4_Cl), which increases the lysosomal pH and thereby slows down autophagic degradation, cleaved GFP accumulates (Figure 6A) [21,27]. Hence, relative abundances of cleaved GFP provide a measure for functional autophagy flux assessments. To see whether the autophagic flux was impaired in *atg-18* and *epg-6* mutant strains, we starved *atg-18(gk378);adIS2122* and *epg-6(bp242);adIS2122* L1 larvae overnight in the presence or absence of NH_4_Cl, and extracted proteins for Western blotting (Figure 6B). Both *atg-18(gk378);adIS2122* and *epg-6(bp242);adIS2122* mutant strains showed a strong accumulation of GFP::LGG-1 and GFP::LGG-1-PE when compared to wild-type (Figure 6B), indicating either a block in autophagy or strong upregulation of GFP::LGG-1 after loss of ATG-18 and EPG-6. Addition of NH_4_Cl caused an accumulation of cleaved GFP in the wild-type due to a block in starvation-induced autophagy as expected, but this was not found in either the *atg-18(gk378);adIS2122* or the *epg-6(bp242);adIS2122* mutant sample (Figure 6B,C; Data S1). The lack of cleaved GFP accumulation following NH_4_Cl treatment in the context of accumulated GFP::LGG-1 and GFP-LGG-1-PE further supports the idea that loss of either ATG-18 or EPG-6 leads to a block in autophagy.

**Figure 7:**
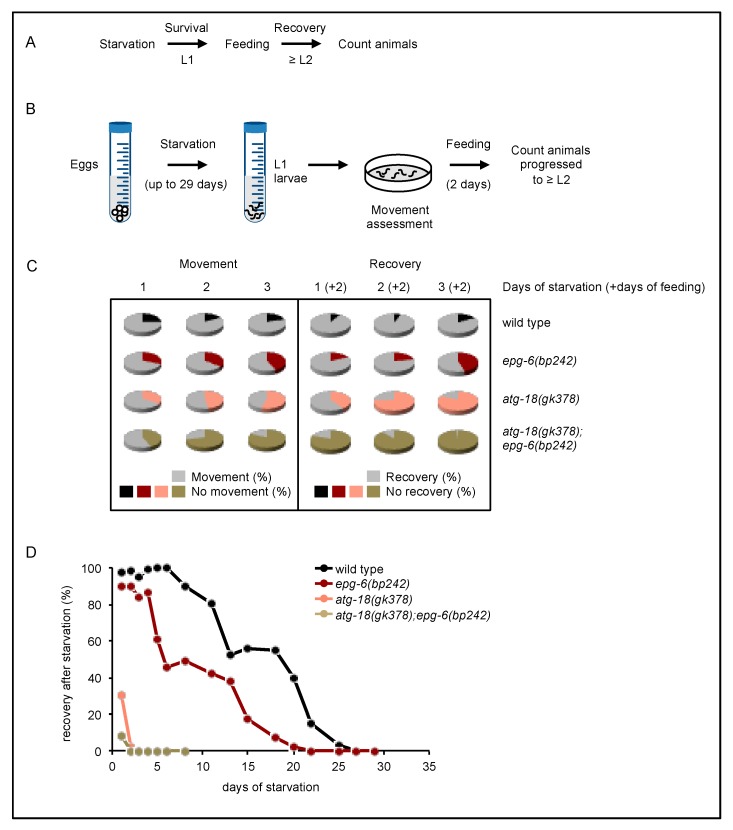
Survival of L1 larvae upon starvation decreased in the absence of ATG-18 and EPG-6. The overall experimental set-up for the survival analysis upon starvation conditions (**A**). L1 larvae hatched in nutrient-free medium and were incubated in this medium for the indicated time. The larvae were then tested for recovery on bacteria-covered plates, while their movement was recorded. Subsequently, the percentage of animals that recovered from starvation in the presence of food was calculated (**B**). The percentages of animals that showed movement upon starvation of 1, 2 or 3 days (**C**, left panel) is shown, as well as the percentages of animals recovering and surviving from the indicated starvation periods in the presence of food: e.g., 1 (+2) stands for 1 day of starvation followed by 2 days of feeding (**C**, right panel). Likewise, over a period of 1–29 days, the percentages of surviving animals were recorded where *atg-18* and/or *epg-6* mutant L1 larvae showed a decreased tolerance to starvation (**D**).

When *C. elegans* eggs hatch in the absence of food, L1 larvae enter an arrested stage, which wild-type larvae survive for up to three weeks and upon re-feeding they recover and develop further [21]. As proper autophagic activity is required for the survival of this starvation period [11,21,29], we aimed to further investigate the physiological roles of ATG-18 and EPG-6 in *C. elegans* by measuring the survival and recovery of L1 larvae upon starvation. (Figure 7). Following synchronization, L1 larvae (*atg-18(gk378)*, *epg-6(bp242)*, and *atg-18(gk378);epg-6(bp242)*) were continuously starved over a period between 1 to 29 days and their survival was assessed by counting larvae that recovered from starvation and progressed through larval stage L2 in the presence of food (Figure 7A,B).

First, we assessed movement abilities of all larvae right after starvation (scheme in Figure 7B, results in Figure 7C, left panel). We found that the majority of wild-type animals showed prominent movement after being starved for 1, 2 or 3 days (Figure 7C, left panel, animals not moving in black) and the majority also recovered from these periods of starvation in the presence of food (Figure 7C, right panel, animals not recovering in black). In contrast, less immediate movement upon 1 to 3 days of starvation was observed in the absence of EPG-6 (Figure 7C, left panel, not moving animals are shown in dark red), as well as reduced survival in the presence of food (Figure 7C, right panel, not recovering animals are shown in dark red). This result is in line with previous reports [10]. Less movement (Figure 7C, left panel, animals not moving in light red) and poorer recovery in the presence of food after starvation periods between 1 to 3 days (Figure 7C, right panel, animals not recovering in light red) was even more prominent in the absence of ATG-18, and severe in the absence of both EPG-6 and ATG-18 (Figure 7C, left panel, not moving animals are shown in gold; right panel, not recovering animals are shown in gold). Moreover, when we assessed the survival of L1 larvae over a longer period (up to 29 days) of starvation (Figure 7D; Data S1), differences in the capacities to recover became even more apparent when comparing wild-type animals with mutant strains deficient for EPG-6 and/or ATG-18 (Figure 7D).

**Figure 8:**
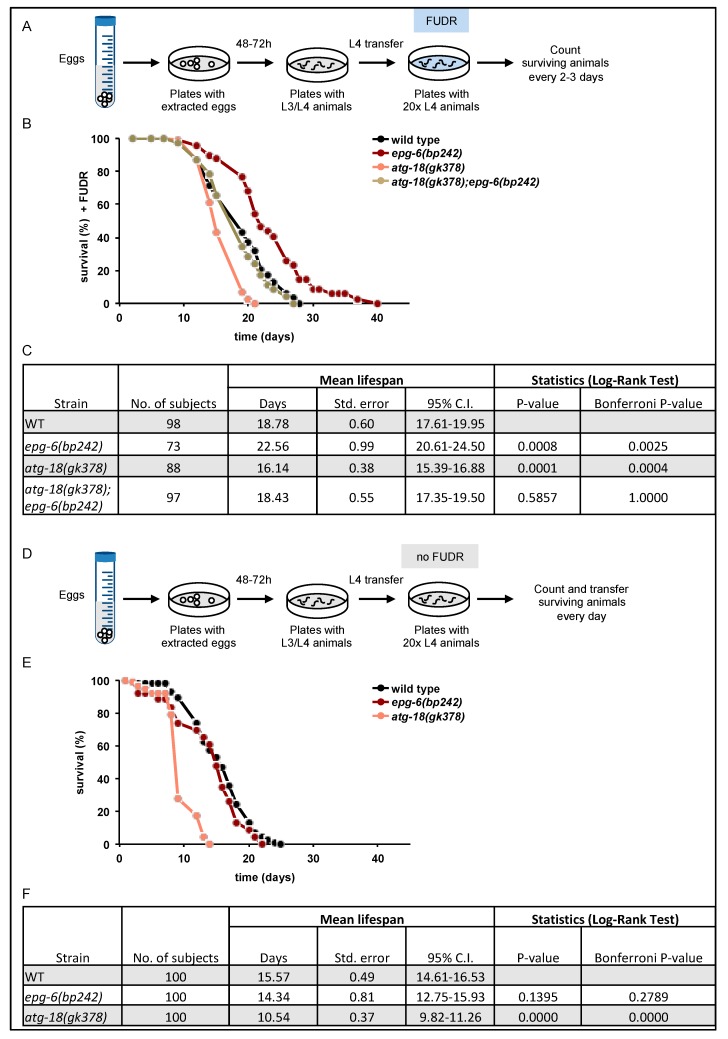
Lifespan increased in the absence of EPG-6. Synchronised animals were grown to L4 larval stage and transferred either to NGM *E. coli* OP50 plates supplemented with FUDR and counted every other day (**A**), or they were counted and transferred each day to fresh NGM/*E. coli* OP50 plates without fluorodeoxyuridine (FUDR) (**D**). The lifespans of wild-type, *atg-18(gk378)*, *epg-6(bp242)*, and *atg-18(gk378);epg-6(bp242)* worms were measured. Survival plot and statistical analysis of one representative set out of three independent experiments is shown (**B**,**C**,**E**,**F**). Statistical analysis was performed using basic survival analysis of the “Online Application for the Survival Analysis of Lifespan Assays Performed in Aging” software. For more information see Data S1.

Functional autophagy and lifespan control was closely correlated in *C. elegans*, and loss of ATG-18 has previously been shown to decrease lifespan [11,12]. However, the role of EPG-6 in adult lifespan regulation had not yet been addressed. Hence, we investigated whether *epg-6* mutant animals would also display a short-lived phenotype in our approach. We performed lifespan assessments by counting living animals on NGM/*E. coli* OP50 plates in the presence of 5-fluorodeoxyuridine (FUDR), a compound that prevents egg hatching (Figure 8A–C). Our results confirmed that *atg-18* mutants have a significantly decreased lifespan (light-red label) compared to the wild-type (black label), (Figure 8B,C). Unexpectedly, *epg-6* mutant worms (dark-red label) lived significantly longer than wild-type worms (Figure 8B,C). Loss of EPG-6 in *atg-18* mutant background (*atg-18(gk378);epg-6(bp242*), gold label) suppressed the short-lived phenotype of *atg-18* mutants and shifted the lifespan curve to wild-type levels (Figure 8B,C). Likewise, downregulation of EPG-6 by RNAi in *atg-18* mutant animals prolonged their lifespan (Figure S2). These results suggest opposing roles for ATG-18 and EPG-6 in lifespan control.

To assess if the compound FUDR may influence the lifespan, we performed lifespan measurements also in the absence of FUDR by transferring animals every day onto fresh plates in order to separate them from their progeny (Figure 8D–F). Under these conditions, loss of EPG-6 function did not extend lifespan when compared to wild-type animals, but also did not show a short-lived phenotype as again seen for *atg-18* mutant animals (Figure 8E,F). These results suggest that the anticipated autophagy-independent role of EPG-6 in lifespan control may be connected to germline function, which is counteracted by the presence of FUDR.

## 4. Discussion

In line with previous observations [10], we provide evidence that in the absence of PROPPIN [4,30] members ATG-18 and EPG-6, both of which are considered to function as PI3P effectors at the nascent autophagosome in *C. elegans* [31,32], autophagosome formation and the autophagic flux was negatively influenced from early embryo throughout all larval stages (L1–4) (Figure 2, Figure 3, Figure 4, Figure 5 and Figure 6). We demonstrated that *epg-6* loss-of-function mutants were less fitted to survive L1 starvation periods [10] and ATG-18 deficient strains had almost completely lost their capability to recover from L1 starvation (Figure 7). Moreover, we found that *epg-6* mutants not only survived starvation better than *atg-18* mutants, but while *atg-18* loss-of-function mutants displayed a short-lived phenotype [11,12], loss of EPG-6 function promoted longevity in *C. elegans* (Figure 8).

By characterizing the *atg-18*, *epg-6* and *atg-18;epg-6* mutant strains we observed high lethality at early larval development (L1) in the absence of ATG-18 (Figure 1), suggesting that ATG-18 is essential even under favourable conditions. To assess the abundance of formed autophagic structures, we measured GFP::LGG-1 puncta formation throughout development of *C. elegans*. In early embryos GFP::LGG-1 puncta count and size decreased in *atg-18* and *epg-6* mutants compared to wild-type (Figure 2), indicating an inhibition of autophagosome formation. It was reported that in *atg-18* mutant embryos small, in *epg-6* mutant embryos enlarged LGG-1 puncta accumulate [10,14,33]; however, we observed enlarged GFP::LGG-1 puncta accumulation of both *atg-18* and *epg-6* mutants in late-stage embryos. The difference to reported studies may be due to the different embryonic stages investigated. As GFP::LGG-1 puncta abundance changes dynamically during embryonic development (see Figure 2, early, late embryos of N2), the developmental stage presents a critical parameter when assessing LGG-1 puncta formation by fluorescence microscopy.

During postembryonic development we observed enlarged GFP::LGG-1 structures both in the head and seam cells of mutant *atg-18* and *epg-6* L1 through L4 larvae; however, in the head, puncta accumulated, while in the seam cells, puncta count decreased (Figure 3, Figure 4 and Figure 5).

Supporting our data, decreased GFP::LGG-1 puncta formation was observed in the seam cells upon *atg-18* RNAi treatment [16] and in *atg-18* mutants [18]. Enlarged GFP::LGG-1 punctate structures in the head and seam cells during larval development suggests that autophagic flux was impaired. In addition, in the seam cells, autophagy initiation might also be inhibited leading to decreased puncta number. In summary, our quantitative assessment of GFP::LGG-1 puncta by fluorescence microscopy showed that aberrant autophagosomal membranes accumulated in the absence of ATG-18 and EPG-6, and in the seam cells, the initiation of autophagosome formation, a feature attributed to a function upstream of LGG-1 (or LC3 in mammals) [10], was also inhibited.

To further investigate whether autophagic flux was impaired, lipidation of GFP::LGG-1 was analyzed by Western blotting and also upon lysosomal inhibition (Figure 6A) [17,21]. Increased LGG-1 and LGG-1-PE levels have been reported for both *atg-18* and *epg-6* mutant strains [10,14,32], which we also observed (Figure 6B). We also showed that cleaved GFP decreased in *atg-18* and *epg-6* mutants, and while cleaved GFP was stabilized by inhibiting lysosomal degradation in wild-type larvae, this was not the case in *epg-6* and *atg-18* mutants (Figure 6B). These results further support the idea that autophagic flux was impaired in the absence of both ATG-18 and EPG-6 in *C. elegans* (Figure 6). 

Consistent with the observation by Lu and colleagues [10], we demonstrated that *epg-6* mutant animals survive starvation in L1 larval stage poorly. Furthermore, we demonstrate that *atg-18* mutant animals tolerate starvation even worse, and in the absence of both ATG-18 and EPG-6 worms cannot survive starvation. Hence, both ATG-18 and EPG-6 are necessary for L1 larvae survival under starvation conditions and for the recovery from L1 starvation periods in the presence of food (Figure 7). As autophagic activity increases in response to starvation in order to degrade polymers and release monomers for recycling purposes, loss of essential autophagy factors, like ATG-18 and EPG-6, likely leads to decreased survival of starvation. Our results support the general concept that autophagy functions as an evolutionarily conserved pathway engaged to secure survival upon starvation, as early demonstrated in autophagy-deficient mice models [1,34].

**Figure 9:**
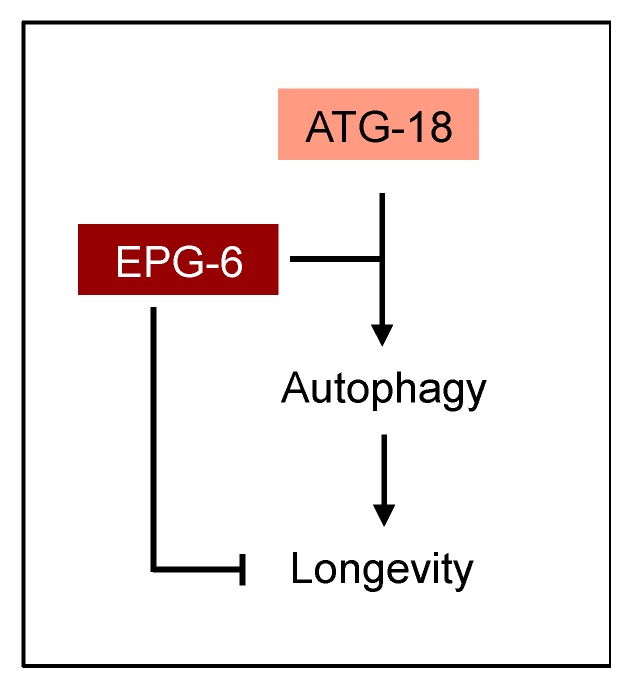
Model for the role of ATG-18 and EPG-6 in autophagy and longevity in *C. elegans*. Both ATG-18 and EPG-6 are considered to function in autophagy as PI3P effectors on the nascent autophagosome, equivalent to the role of their homologues in mammals [7]. In this context, EPG-6 is considered to function downstream of ATG-18 during autophagosome formation [7,10]. A novel, autophagy-independent role of EPG-6 is postulated in lifespan control, which is likely connected to germline signaling.

Autophagy has an essential role in lifespan regulation as impairment of autophagy is connected to decreased lifespan, hence, autophagy is considered an anti-ageing and pro-survival mechanism [35]. Our results provide evidence that ATG-18 and EPG-6 have opposing roles in lifespan control (Figure 9). 

According to previous reports, we observed decreased lifespan in the absence of ATG-18 [3], while in the absence of EPG-6 we found that lifespan was significantly increased and *atg-18;epg-6* double-mutants lived also longer than *atg-18* worms (Figure 8). As autophagy is considered to counteract ageing, and autophagic degradation is impaired in the absence of EPG-6, we suggest that the lifespan promoting effect of EPG-6 is autophagy-independent. As ATG-18 was recently reported to regulate lifespan cell non-autonomously [17,18], it is of interest to assess in future studies whether EPG-6 may control lifespan in *C. elegans* independent of its role in autophagy, also in a cell non-autonomous manner. As increased lifespan upon knockdown of some autophagy genes was reported before [13], it is of intense interest to assess autophagy-dependent and autophagy-independent functions of ATGs in lifespan control, and also, whether autophagy may promote ageing under some conditions.

Interestingly, our lifespan analyses revealed an FUDR-dependent lifespan regulating mechanism in the absence of EPG-6 (Figure 8). Fluorodeoxyuridine (FUDR) is a thymidylate synthase inhibitor, which inhibits DNA synthesis in developing embryos. In early publications, it was reported that FUDR allows synchronization without interfering with the lifespan of *C. elegans* [36]; however, recent publications suggest that FUDR can prolong the lifespan of both wild-type and certain mutant animals [37,38,39]. Our experiments support this observation, as FUDR-treated wild-type animals indeed showed extended lifespan compared to untreated animals (Figure 8) and we also observed lifespan extension of *epg-6* mutants upon FUDR treatment. In this context, it is of great interest that FUDR was demonstrated to activate stress response pathways [40]. With this notion taken into consideration, it may be that the long-lived phenotype observed in the absence of EPG-6 function is provoked by FUDR conferring stress conditions, and that EPG-6’s loss-of-function under non-stress conditions would not influence lifespan, which in fact was found when we assessed the lifespan of *epg-6* mutant animals in the absence of FUDR. In fact, several ATG mutant strains have been identified where the expected short-lived phenotype was not present under non-stress conditions [16,41,42]. 

It also bears mentioning that germline ablation increases the lifespan of *C. elegans* in a DAF-2/DAF-16 dependent manner [43], and increased lifespan upon FUDR treatment was reported for FEM-3, a protein involved in oogenesis [38]. As the lifespan extension of the *epg-6* mutant animals was observed under FUDR treatment conditions, it is tempting to speculate that the autophagy-independent function of EPG-6 could likely be found in the germline, as this is a prominent pathway affected by FUDR treatment. Here, EPG-6 may have a lifespan-repressing function as part of the overall ageing program. 

Autophagy-independent functions of ATG proteins, as here suggested for EPG-6 in lifespan control, have been reported for several ATG proteins [44,45,46,47]. In fact, the serine-/threonine-specific protein kinase ULK1, UNC-51 in *C. elegans*, activated upon energy or nutrient deprivation in order to induce autophagy via signaling through the PI3KC3 [1,2,45], was originally identified as being required for extending axons along the dorsoventral axis in *C. elegans* [48]. The UNC-51 protein interacts with UNC-14 [49,50] and regulates the intracellular localization of the netrin receptor UNC-5 in *C. elegans* [51], thereby controlling axon guidance [52] independent of UNC-51’s role in autophagy [46,53,54]. Moreover, it has been reported that constitutive autophagy in the central nervous system in mice is executed in the absence of *Ulk1* (and *Ulk2*) [46], a process referred to as non-canonical autophagy, whereby autophagy proceeds in the absence of canonical ATG proteins [44]. In this context, it was demonstrated that Ulk1/UNC-51 regulates ER-to-Golgi trafficking [46]. Further autophagy-independent functions of ATG proteins have been described. By way of example, ATG7, a critical member of two ubiquitin-like conjugation systems in autophagy and required for LC3 lipidation [1,45], was shown to positively regulate the expression of cell cycle arrest genes in complex with the tumor suppressor p53 and in response to metabolic stress in mice [55]. Further, it was also found that ATG16L1, functioning in association with ATG5-ATG12 in promoting the lipidation of LC3 at autophagosomal membranes [1,45] can also promote LC3 lipidation at endolysosomal membranes during non-canonical autophagy [56]. 

The examples above refer to autophagy-independent functions of ATG proteins, where it has been shown that homologous proteins in different species carry out equivalent biological functions. With regard to the role of ATG-18 and EPG-6, this assignment still awaits clarification. Whereas *C. elegans* expresses the two PROPPIN members ATG-18 and EPG-6, three PROPPIN members, Atg18, Atg21 and HSV2, have been identified in yeast, and the four WIPI proteins (WIPI1 through WIPI4) present the PROPPIN members in humans [4]. *Caenorhabditis elegans* ATG-18 belongs to one paralogous group of the PROPPIN family together with yeast Atg18 and Atg21, and human WIPI1 and WIPI2. *Caenorhabditis elegans* EPG-6 belongs to the other PROPPIN group together with yeast HSV2 and human WIPI3 and WIPI4 [8]. Based on this phylogenetic relationship it can be anticipated that PROPPIN members belonging to the same paralogous group share equivalent biological functions. This, however, seems different with regard to the PROPPIN family as follows. The ancestor of the PROPPIN family, Atg18 in *Saccharomyces cerevisiae* [57], is essential for autophagy and interacts with Atg2 [57,58,59] for tethering the forming autophagosome to the endoplasmic reticulum in yeast [60]. Although in *C. elegans*, ATG-18 also fulfills an essential role in autophagy, its paralogue EPG-6 was shown to interact with ATG-2 in order to control autophagosome expansion downstream of ATG-18 [10]. This has also been shown for human WIPI4 that specifically binds to ATG2 and controls the expansion of the nascent autophagosome, downstream of WIPI1 and WIPI2 [7]. Hence, the ancestral yeast Atg18 function is replicated in EPG-6 in *C. elegans* and in WIPI4 in humans. Our results here point out that ATG-18 and EPG-6 should also fulfill non-redundant roles in lifespan regulation, as in *C. elegans*, both are required for autophagy which is considered to counteract ageing. In future studies it will be of interest to decipher the differential contribution of PROPPIN members in lifespan regulation, not only in *C. elegans*, but also in other model organisms, including mice. 

## Supplementary Materials

The following are available online at https://www.mdpi.com/2073-4409/8/3/236/s1. Supplementary Figures S1 and S2. Supplementary Table S1—*C. elegans* strains used in this study. Supplementary Data 1—Extended Data for experiments shown in Figures 2–8, Supplementary Figures S1 and S2.
