# Supplementary material for: ATG-18 and EPG-6 are Both Required for Autophagy but Differentially Contribute to Lifespan Control in Caenorhabditis elegans
Source: Cells. 2019 Mar 12;8(3):236. doi: 10.3390/cells8030236 (PMC6468378; doi:10.3390/cells8030236)
Supplement: Supplementary file 1 [file cells-08-00236-s001.zip › cells-448378-final-suppl/cells-448378-final-suppl.pdf]

ATG-18 and EPG-6 are Both Required for Autophagy but Differentially Contribute to Lifespan Control in *Caenorhabditis elegans*

Zsuzsanna Takacs [1,2], Katharina Sporbeck [1,2], Jennifer Stoeckle [1], Maria Jhaneth Prado Carvajal [1], Mona Grimmel [1] and Tassula Proikas-Cezanne [1,2]*

[1] Department of Molecular Biology, Interfaculty Institute of Cell Biology, Eberhard Karls University Tuebingen, Germany;
[2] International Max Planck Research School 'From Molecules to Organisms', Max Planck Institute for Developmental Biology and Eberhard Karls University Tuebingen, Germany
* Correspondence: tassula.proikas-cezanne@uni-tuebingen.de; Phone: +49-7071-2978895; Fax: +49-7071-295359.

Supplementary Material

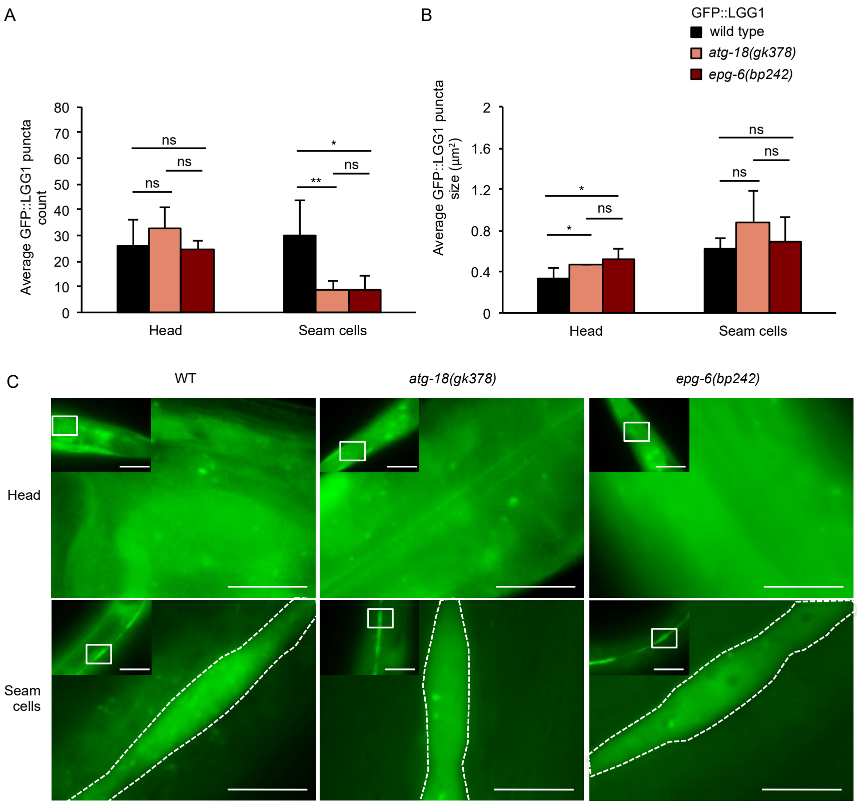

Supplementary Figure 1. – GFP::LGG-1 puncta accumulate in adult nematodes in the head but decrease in numbers in seam cells. GFP::LGG-1 puncta formation was analysed in the head and seam cells of wildtype, *atg-18(gk378)*, and *epg-6(bp242)* adult animals expressing the *adIS2122* transgene. Fluorescence microscopy images were quantified using the stress granule counter plugin of ImageJ. The average numbers (A) and size (B) of GFP::LGG-1 puncta were calculated. Representative images are shown (C). Scale bar: 20 µm. Significances calculated using the Kruskal-Wallis-Test, * P<0.05, ** P<0.01. Supplementary material is available (Suppl. Data 1).

A

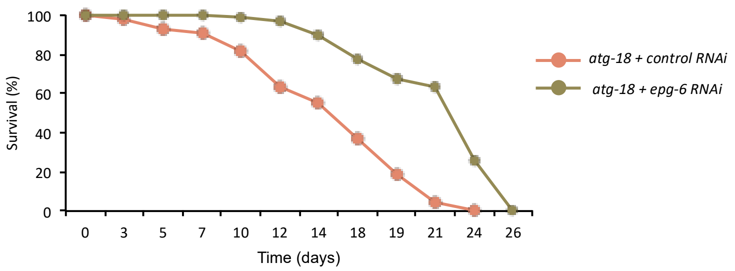

RNAi	No. of subjects	Mean Lifespan			Statistics (Log-Rank Test)	
		Days	Std. error	95% C.I.	P-value	Bonferroni P-value
contr. RNAi	98	15.55	0.68	14.21-16.89		
epg-6 RNAi	101	22.05	0.60	20.87-23.23	0.0E+0	0.0E+0

Supplementary Figure 2. – Lifespan increases in the absence of EPG-6. Synchronised *atg-18(gk378)* animals were grown to L4 larval stage and transferred to NGM/FUDR plates covered with control or epg-6 targeting RNAi bacteria and counted every other day (A). Survival plot and statistical analysis are shown. Statistical analysis was performed using basic survival analysis of the "Online Application for the Survival Analysis of Lifespan Assays Performed in Aging" software. Supplementary material available: Suppl. Data 1.

Supplementary Table 1 – *C. elegans* strains used in this study.

Strain	Genotype	Laboratory (origin)	Ref.	Outcrossed	Obtained from	Results shown here in
N2	Bristol wild isolate	J. Hodgkin	[20]	/	CGC	Figures 1,7,8
DA2123	*adIs2122 [lgg-1p::GFP::lgg-1 + rol-6(su1006)]*	L. Avery	[22]	7x	CGC	/
VC893	*atg-18(gk378) V*	D. Moerman	[12]	1x	CGC	Figure 1
HZ1690	*epg-6(bp242) III; him-5(e1490) V*	H. Zhang	[10]	2x	CGC	Figure 1
TPC8	*epg-6(bp242) III; him-5(e1490) V*		/	9x	This study	Figures 1,7,8
TPC17	*atg-18(gk378) V*		/	4x	This study	Figures 1,7,8, Suppl. Figure 2
TPC22	*adIs2122 [lgg-1p::GFP::lgg-1 + rol-6(su1006)]*		/	10x	This study	Figures 2,3,4,5,6, Suppl. Figure 1
TPC32	*epg-6(bp242) III; him-5(e1490) V; lgg-1::GFP+rol-6(su1006)*		/	10x	This study	Figures 2,3,4,5,6
TPC34	*atg-18(gk378) V; lgg-1::GFP+rol-6(su1006)*		/	10x	This study	Figures 2,3,4,5,6
TPC12	*atg-18(gk378) V; epg-6(bp242) III; him-5(e1490) V*		/	5x;6x	This study	Figures 1,7,8
